# Citrus Naringenin Increases Neuron Survival in Optic Nerve Crush Injury Model by Inhibiting JNK-JUN Pathway

**DOI:** 10.3390/ijms23010385

**Published:** 2021-12-29

**Authors:** Jie Chen, Hui Li, Changming Yang, Yinjia He, Tatsuo Arai, Qiang Huang, Xiaodong Liu, Linqing Miao

**Affiliations:** 1 Beijing Advanced Innovation Center for Intelligent Robots and Systems, Beijing Institute of Technology, Beijing 100081, China; jiechen@pku.edu.cn (J.C.); changmyang@bit.edu.cn (C.Y.); tarai118@bit.edu.cn (T.A.); qhuang@bit.edu.cn (Q.H.); 2 School of Life Sciences, Peking University, Beijing 100871, China; 3 Beijing Research Institute of Chinese Medicine, Beijing University of Chinese Medicine, Beijing 100029, China; lhuibucm916@163.com (H.L.); heyinjiaKate@icloud.com (Y.H.); 4 School of Mechatronical Engineering, Beijing Institute of Technology, Beijing 100081, China

**Keywords:** JNK-JUN pathway, JUN phosphorylation, naringenin, neuroprotection, optic nerve crush, traumatic nerve injury, retinal ganglion cells

## Abstract

Traumatic nerve injury activates cell stress pathways, resulting in neuronal death and loss of vital neural functions. To date, there are no available neuroprotectants for the treatment of traumatic neural injuries. Here, we studied three important flavanones of citrus components, in vitro and in vivo, to reveal their roles in inhibiting the JNK (c-Jun N-terminal kinase)-JUN pathway and their neuroprotective effects in the optic nerve crush injury model, a kind of traumatic nerve injury in the central nervous system. Results showed that both neural injury in vivo and cell stress in vitro activated the JNK-JUN pathway and increased JUN phosphorylation. We also demonstrated that naringenin treatment completely inhibited stress-induced JUN phosphorylation in cultured cells, whereas nobiletin and hesperidin only partially inhibited JUN phosphorylation. Neuroprotection studies in optic nerve crush injury mouse models revealed that naringenin treatment increased the survival of retinal ganglion cells after traumatic optic nerve injury, while the other two components had no neuroprotective effect. The neuroprotection effect of naringenin was due to the inhibition of JUN phosphorylation in crush-injured retinal ganglion cells. Therefore, the citrus component naringenin provides neuroprotection through the inhibition of the JNK-JUN pathway by inhibiting JUN phosphorylation, indicating the potential application of citrus chemical components in the clinical therapy of traumatic optic nerve injuries.

## 1. Introduction

Traumatic neural injuries always lead to immediate neural degeneration and subsequent neuronal death. To date, there are no available neuroprotectants for the treatment of traumatic neural injuries. The immature fruit of *Citrus aurantium* L., named Zhishi, is used in traditional Chinese medicine [1] for its various therapeutic effects, such as cardioprotective, hepatoprotective, and anti-inflammatory effects [2,3]. Naringenin, hesperidin, and nobiletin are flavanones that are widely detected in most citrus species, including Zhishi.

Naringenin (4′,5,7-trihydroxyflavanone, with the molecular formula C_15_H_12_O_5_ and a molecular weight of 272.25 g/mol) is a plant bioflavonoid that is naturally found in citrus fruits (up to 10% of its dry weight) [4], it contains two benzene rings linked together with a heterocyclic pyrone ring [5,6]. Hesperidin (hesperetin 7-rutinoside), a flavanone glycoside, is isolated from citrus fruit with a content of about 3%, and has the molecular formula C_28_H_34_O_15_ and a molecular weight of 610.56 g/mol [7,8]. Nobiletin (3′,4′,5,6,7,8-hexamethoxyflavone, with the molecular formula C_21_H_22_O_8_ and a molecular weight of 402.39 g/mol) is a poly-methoxylated flavone that originates from citrus peel, with a content of about 0.08% [9]. It has been reported as a retinoid acid receptor-related orphan receptors (RORs) agonist [10], with six methoxy groups on its flavone skeleton distributed at the 5, 6, 7, 8-positions on the A-ring and 3′, 4′-positions on the B-ring. These chemical components are reported to have neuroprotective effects in neural degeneration [11,12]. Recent studies show that traumatic optic nerve injury, such as optic nerve crush (ONC), can induce the activation of endoplasmic reticulum (ER) stress pathways [13], and an important cell stress pathway, the JNK (c-Jun N-terminal kinase)/JUN pathway [14], resulting in serious neurodegeneration and death of retinal ganglion cells (RGCs) [13,15].

JNK, also known as stress-activated protein kinase (SAPK), is preferentially activated by a variety of environmental stresses. JNK activity is higher in the brain than in any other tissues [16,17]. There are three JNK genes, each of which undergoes alternative splicing, resulting in numerous isoforms [18]. JNKs are important components of the classical mitogen-activated protein kinase (MAPK) signaling cascade. Stress signals are delivered to this cascade by small GTPases of the Rho family (Rac, Rho, cdc42) [18]. JNKs, when activated and forming a dimer, translocate to the nucleus and regulate transcription through c-Jun and other transcription factors [18,19]. It is reported that *Jun* knockout significantly increased RGC survival [20,21]. Our previous study showed that *Chop* (the key pro-apoptotic transcription factor of the ER stress pathway) knockout also increased RGC survival [22,23]. However, a study reported that *Jun* knockout showed better RGC survival (93%) than *Chop* knockout (51%) [24]. Therefore, the JNK-JUN pathway plays a more significant role than the ER stress pathway in injury-induced neural death.

All these neuroprotective effects in traumatic optic nerve injury models are based on transgenic knockout mice; however, this transgenic method is not practical in the clinic. Therefore, it is urgent and necessary to look for safe chemical compounds to provide neuroprotection after traumatic optic nerve injury in the clinic. Natural chemical compounds from citrus food are the best candidates for fulfilling the requirements of both safe and potential neuron neuroprotection. Citrus components are reported to have neuroprotective effects, however, whether these components can regulate the JNK-JUN pathway and increase RGC survival after traumatic nerve injury remains elusive. In this study, we investigated the roles of three important components of Citrus flavanones—naringenin, hesperidin, and nobiletin, in the neuroprotection of RGCs in traumatic ONC injury mouse models, and revealed the underlying molecular mechanisms of their neuroprotective effects in regulating the JNK-JUN pathway.

## 2. Results

### 2.1. Optic Nerve Injury Activated JNK-JUN Pathway and Caused RGC Death

**Figure 1:**
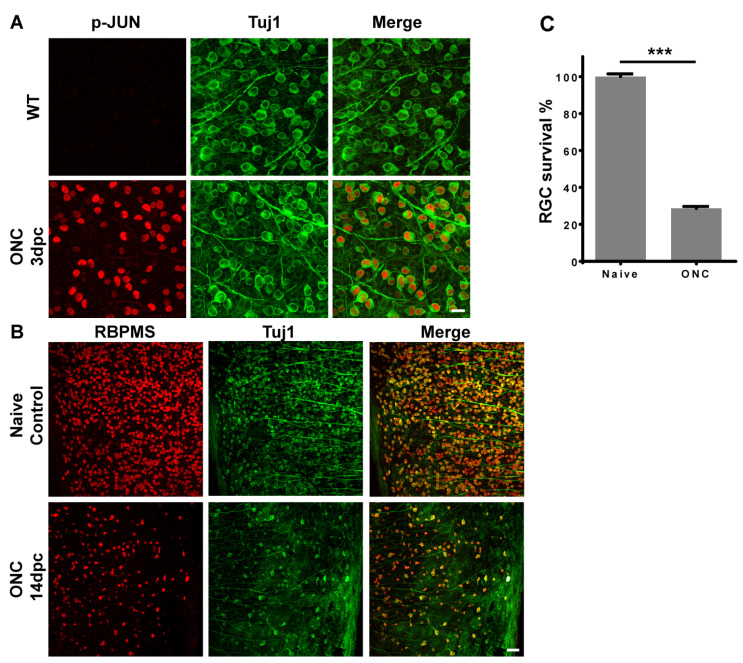
ONC injury increases JUN phosphorylation and results in RGC death. (**A**) Representative images of whole retina co-immunostained with antibodies against phospho-JUN and βIII tubulin (Tuj1), scale bar: 20 μm; (**B**) Representative images of whole retina co-immunostained with antibodies against RBPMS (RGC marker) and βIII tubulin (Tuj1), scale bar: 50 μm; (**C**) Statistical analysis of RGC survival 14 days after ONC injury. Data are represented as mean ± SEM, *n* = 9; *** *p* < 0.001; 3 dpc: 3 days post-crush; 14 dpc: 14 days post-crush; ONC, optic nerve crush; RGCs, retinal ganglion cells.

To identify whether the JNK-JUN pathway is activated after traumatic ONC injury, we generated traumatic ONC injury mouse models and verified the phosphorylation level of JUN. Three days after ONC, we co-stained the whole retina with an antibody against phosphorylated JUN and a neuron marker-βIII tubulin (Tuj1). The results showed that the phosphorylation of JUN in RGCs was significantly increased after ONC injury compared with no JUN phosphorylation in wild type (WT) retinas (Figure 1A). We then examined the survival of RGCs at 14 days post-crush (dpc) by staining the retina with an RGC-specific marker, RBPMS (RNA binding protein with multiple splicing). Imaging and RGC counting results showed that the survival rate of RGCs was significantly decreased after injury, with a survival rate of 29% (Figure 1B,C).

### 2.2. Effects of Citrus Components on Inhibiting Stress Induced Pro-Apoptotic JNK-JUN Pathway

**Figure 2:**
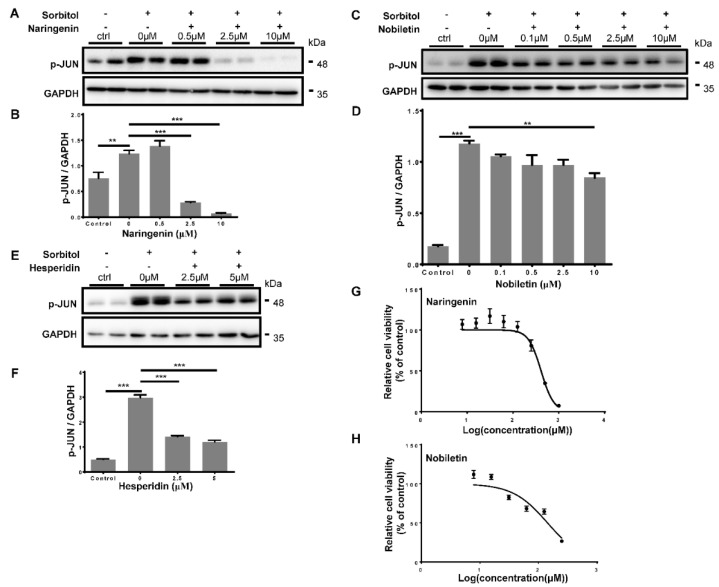
Inhibition of stress-induced pro-apoptotic JNK-JUN pathway by citrus components in HEK293T cells. (**A**) Western blot results of treatments of different concentrations of naringenin, after pre-incubation with sorbitol to activate the JNK-JUN pathway in HEK293T cells. (**B**) Statistical analysis of phosphorylation level of JUN relative to GAPDH loading control after treatments of different concentrations of naringenin. (**C**) Western blot results of treatments of different concentrations of nobiletin, after pre-incubation with sorbitol to activate the JNK-JUN pathway in HEK293T cells. (**D**) Statistical analysis of phosphorylation level of JUN relative to GAPDH loading control after treatments of different concentrations of nobiletin. (**E**) Western blot results of treatments of different concentrations of hesperidin, after pre-incubation with sorbitol to activate JNK-JUN pathway in HEK293T cells. (**F**) Statistical analysis of phosphorylation level of JUN relative to GAPDH loading control after treatments of different concentrations of hesperidin. Rabbit anti-phospho-JUN antibody was used to detect JUN phosphorylation. Mouse anti-GAPDH antibody was used to detect loading control-GAPDH. Data are represented as mean ± SEM of 4 replicates; ** *p* < 0.01; *** *p* < 0.001. (**G**) Relative cell viability analysis of naringenin in HEK 293T cells. (**H**) Relative cell viability analysis of nobiletin in HEK 293T cells. Each point is represented as mean ± SEM of 6 replicates.

Since optic nerve injury activated the pro-apoptotic JNK-JUN pathway and resulted in neuronal death, to assess whether citrus components could inhibit the JNK-JUN pathway in vitro, we induced the pro-apoptotic JNK-JUN pathway in HEK293T cells and performed a series of concentration tests of the citrus components. HEK293T cells were incubated with 500 mM sorbitol for 30 min to activate the JNK-JUN pathway and induce the phosphorylation of JUN [25]. Western blot results showed that the phosphorylation level of JUN was significantly increased after sorbitol incubation (Figure 2A); 0.5 μM naringenin treatment for 1 h after sorbitol incubation did not affect JUN phosphorylation, while 2.5 μM naringenin treatment significantly decreased JUN phosphorylation and 10 μM naringenin treatment completely blocked JUN phosphorylation (Figure 2A,B). We then examined the effect of nobiletin on the inhibition of JUN phosphorylation. We tested four concentration gradients of nobiletin: 0.1 μM, 0.5 μM, 2.5 μM, and 10 μM; western blot results showed that 0.1–2.5 μM nobiletin incubation for 1 h was able to decrease the phosphorylation level of JUN but it was not significant (Figure 2C,D). When nobiletin concentration was increased up to 10 μM there was a significant inhibition of JUN phosphorylation; however, the inhibition effect was mild compared to naringenin treatment (Figure 2B,D). Next, we assessed the inhibitory effects of hesperidin treatment. Western blot results showed that 2.5 μM hesperidin treatment for 1 h decreased the phosphorylation level of JUN; however, increasing the concentration of hesperidin could not further decrease the JUN phosphorylation level (Figure 2E,F). Cell toxicity analysis showed that the concentration of 50% inhibition (IC_50_) of naringenin and nobiletin in HEK 293T cells was 409.6 μM and 142.4 μM (Figure 2G,H), respectively, which were much higher than the working concentrations in JNK-JUN inhibition experiments (Figure 2A,C). Due to the extremely low solubility of hesperidin in cell medium, we failed to obtain the IC_50_ of hesperidin (data not shown). Therefore, all three citrus components were able to inhibit stress-induced JUN phosphorylation, with naringenin exhibiting the best inhibitory effect.

### 2.3. Effects of Citrus Components on RGC Survival after Optic Nerve Injury

**Figure 3:**
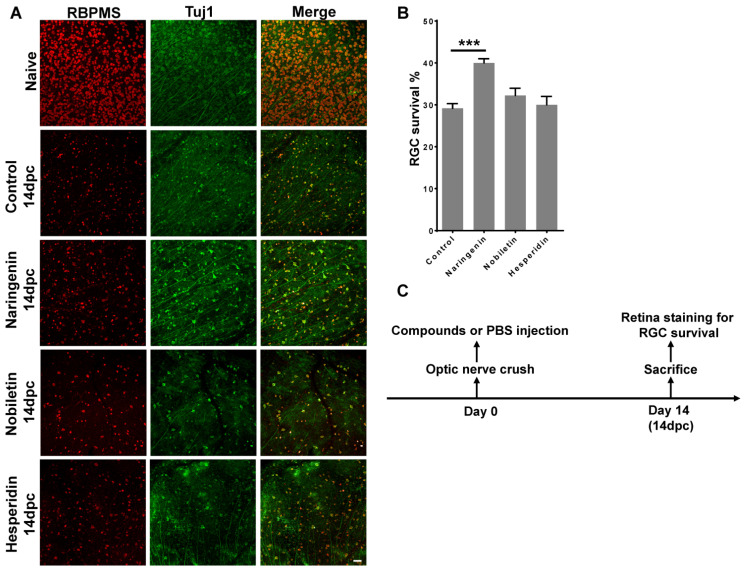
Neuroprotection effects of RGCs in traumatic optic nerve injury model after treatment with citrus components. (**A**) Representative images of naïve retina, 14 dpc retina, and 14 dpc retina after treatment with naringenin, nobiletin or hesperidin, respectively. Retinas were co-immunostained with rabbit antibody against RGC specific marker-RBPMS and mouse antibody against neuron specific marker-βIII tubulin (Tuj1). Scale bar: 50 μm. (**B**) Statistical analysis of percentage of survived RGCs in groups treated with compounds relative to naive control retina. Data are represented as mean ± SEM, *n* = 7; *** *p* < 0.001. (**C**) The chronogram of the mouse experiments for RGC survival study. 14 dpc: 14 days post-crush; RGCs, retinal ganglion cells.

Since all three citrus components were able to inhibit JUN phosphorylation in vitro, we examined whether they had neuroprotective effects in traumatic ONC injury mouse models. We labeled the retinal RGCs with an antibody against the RGC-specific marker RBPMS. Results showed that at 14 dpc, the control injured retina showed a survival of 29% RGCs compared to naive retinas. However, after injection of 2 μL of 10 μM naringenin into the vitreous chamber of the eyeball after ONC injury, the RGC survival rate was significantly increased to 40% (Figure 3A,B), which was consistent with the western blot results in which 10 μM naringenin treatment completely inhibited JUN phosphorylation (Figure 2A,B). We then evaluated the neuroprotective effect of nobiletin in the ONC injury mouse models. Retina immunostaining results showed that 10 μM nobiletin treatment did not increase RGC survival compared with that of the control of ONC injury alone (Figure 3A,B). Finally, we evaluated the neuroprotective effects of hesperidin in ONC mice. Retina immunostaining results showed that 10 μM hesperidin treatment had no effect on increasing RGC survival compared with that of the control of ONC injury alone (Figure 3A,B); hesperidin treatment had modestly inhibited stress-induced JUN phosphorylation (Figure 2E,F). Therefore, in vivo tests of all three citrus components in the ONC mouse models revealed that only naringenin treatment had a neuroprotective effect on RGC after injury (Figure 3B). The scheme showed the chronogram of mouse experiments for studying RGC survival after ONC (Figure 3C).

### 2.4. Naringenin Promoted RGC Survival by Inhibiting JNK-JUN Pathway In Vivo

**Figure 4:**
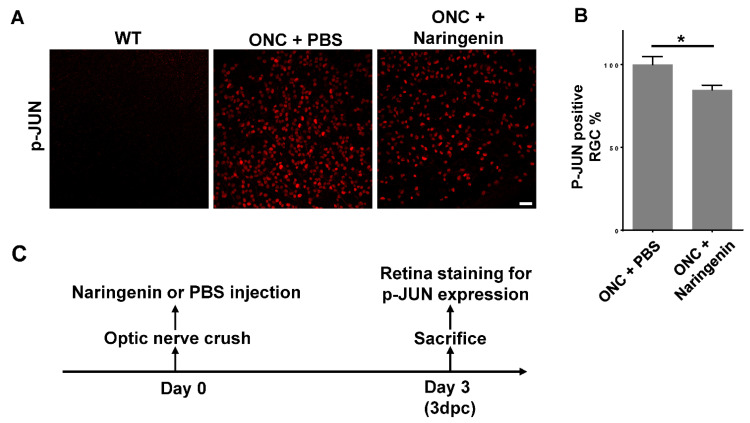
Naringenin promotes RGC survival by inhibiting JNK-JUN pathway. (**A**) Representative images of WT retina, ONC retina treated with PBS control, and ONC retina treated with naringenin. Retinas were immunostained with antibody against phospho-JUN, scale bar: 50 μm. (**B**) Statistical analysis of p-JUN positive RGC number in naringenin treatment group relative to the control retina of PBS treatment. Data are represented as mean ± SEM, *n* = 8; * *p* < 0.05. (**C**) The chronogram of the mouse experiments for studying the inhibition of JNK-JUN pathway. 3 dpc: 3 days post-crush; RGCs, retinal ganglion cells.

Since naringenin was able to promote RGC survival after ONC, we then examined whether the neuroprotective effect of naringenin was fulfilled through inhibiting the JNK-JUN pathway in vivo. We injected 2 of 10 μM naringenin into the vitreous chamber of the mouse eyeball after ONC, and stained the whole retina with an antibody against phosphorylated JUN. The results showed that the p-JUN positive RGC number was significantly decreased in ONC retina with naringenin treatment compared with ONC retina with control PBS treatment (Figure 4A), and the percentage of p-JUN positive RGCs was decreased by around 15% (Figure 4B). The inhibition of the JNK-JUN pathway by naringenin in vivo was consistent with the result of HEK 293T cells in vitro (Figure 2A). The scheme showed the chronogram of mouse experiments for studying the inhibition of the JNK-JUN pathway in vivo after ONC (Figure 4C).

## 3. Discussion

In this study, we first showed that all three citrus components inhibited stress-induced JUN phosphorylation, and among the three components, naringenin had the best JUN phosphorylation inhibition effect. All three compounds inhibited JUN phosphorylation in HEK 293T cells, however, only naringenin showed neuroprotective effects in vivo, increasing the RGC survival in traumatic ONC injury mouse models. Further JUN phosphorylation study in ONC retina with naringenin treatment confirmed the inhibition of JNK-JUN pathway by naringenin in RGCs. Previous studies have also reported that all three citrus components play protection roles in cultured cell lines [26]. These studies indicate their potential roles in neuroinflammation inhibition and neuroprotection in vivo. Traumatic optic nerve injuries activate both the ER stress pathway and JNK-JUN pathway, resulting in an increase in the expression of pro-apoptotic transcription factors of *Ddit3* (also known as *Chop*) and *Jun* [13]. Other research groups have also reported that the JNK-JUN pathway was activated and the phosphorylation level of JUN was increased after ONC injury [27,28], consistent with the results of our observation in traumatic ONC model. A previous study had demonstrated that 14 days after ONC, *Ddit3* deficient mice showed 51% RGC survival, whereas *Jun* deficient mice showed 93% RGC survival, compared with control 29% RGC survival in ONC alone mice. *Jun* deficiency showed an overwhelming neuroprotective effect compared to that of *Ddit3* deficiency [24].

Whether Citrus compounds can inhibit the JNK-JUN pathway and whether the inhibition of JUN phosphorylation still have the same neuroprotective effect as *Jun* knockout are still unknown. Therefore, in this study, we evaluated the neuroprotective effect of citrus components via inhibition of JUN phosphorylation. Due to the superior neuroprotective effect of *Jun* deficiency, we focused on studying the effects of the three citrus components on the inhibition of JUN phosphorylation. Although the in vitro study demonstrated that naringenin, nobiletin, and hesperidin were all able to inhibit JUN phosphorylation, only naringenin showed neuroprotection in in vivo experiments. Further study revealed that only naringenin inhibited JUN phosphorylation in ONC RGCs, while the other two compounds had no inhibition effects in vivo (data not shown). Considering the complete inhibition of JUN phosphorylation by naringenin and partial inhibition by the other two compounds in HEK 293T cells, naringenin still had inhibition effects on JUN phosphorylation in vivo. The weakened effects of p-JUN inhibition by these compounds may be due to the complicated environment in vivo where the JNK-JUN pathway may be affected by many other factors. Previous studies have shown a substantial increase in RGC survival after crush injury; however, all these neuroprotection studies were based on transgenic mice with gene knockout or overexpression [22,24,29,30]. This kind of pre-treatment or pre-protection is not practical under clinical circumstances. Patients do not receive treatment until they are injured; therefore, there is an urgent need to search for a safe neuroprotectant that can provide neuroprotection after neural injury. The in vivo neuroprotective effects of naringenin treatment are not comparable to those of transgenic knockout mice; however, in this study, the compound was delivered after traumatic ONC injury, which perfectly mimicked the situation in clinical practice. Moreover, drug delivery is more convenient and simpler than gene editing for clinical applications. Furthermore, we can modify the chemical structure of naringenin or attempt different combinations of chemical compounds in future studies to achieve better neuroprotective effects.

In conclusion, our study demonstrated that the three flavanone components of citrus extract inhibited the stress-induced phosphorylation of JUN, with complete inhibition by naringenin and partial inhibition by nobiletin and hesperidin. Experiments using traumatic ONC injury mouse models demonstrated that naringenin showed a significant neuroprotective effect by increasing RGC survival after neural injury, and the neuroprotection effect of naringenin in vivo was fulfilled by inhibiting the JNK-JUN pathway in injured RGCs.

## 4. Materials and Methods

### 4.1. Animals

We performed the experiments in 6-weeks-old C57BL/6J mice. All animal experiments were conducted in accordance with the National Institutes of Health Guide for the Care and Use of Laboratory Animals. The animal study was reviewed and approved by the Institutional Animal Care and Use Committee of Beijing Institute of Technology and Peking University.

### 4.2. Reagents

Naringenin (Cat# W530098, Sigma-Aldrich, Saint Louis, MO, USA), nobiletin (Cat# 92600, Sigma-Aldrich), hesperidin (Cat# 50162, Sigma-Aldrich), mouse neuronal class β-III tubulin (clone Tuj1, 1:400, Cat# MMS-435P, Biolegend, San Diego, CA, USA), rabbit anti-RBPMS (1:1000, Cat# GTX118619, GeneTex, Irvine, CA, USA), rabbit anti-p-JUN (1:200, Cat# 3270, Cell Signaling Technology, Danvers, MA, USA), mouse anti-GAPDH (clone 5A12, 1:1000, Cat# DE0621, BIODEE, Beijing, China), Cy2/Cy3-conjugated antibodies (1:200; Cat# 115-225-166/112-165-167, Jackson ImmunoResearch Inc, West Grove, PA, USA), Fluoromount-G (Cat# 0100-01, SouthernBiotech, Birmingham, AL, USA), phosphate buffered saline (PBS) (Cat# P3813, Sigma-Aldrich), Tris HCl (Cat# 15506017, Invitrogen, Carlsbad, CA, USA), NaCl (Cat# S3014, Sigma-Aldrich), sodium dodecyl sulfate (SDS) (Cat# 28364, Thermo Scientific, Waltham, MA, USA), NP-40 (Cat# 28324, Thermo Scientific), sodium deoxycholate (Cat# 89904, Thermo Scientific), sodium pyrophosphate (Cat# 221368, Sigma-Aldrich), sodium fluoride (Cat# 201154, Sigma-Aldrich), sodium orthovanadate (Cat# S6508, Sigma-Aldrich), protease inhibitor cocktail (Cat# 87786, Thermo Scientific), paraformaldehyde (Cat# P6148, Sigma-Aldrich), goat serum (Cat# 16210072, GIBCO, Carlsbad, CA, USA), Triton X-100 (Cat# HFH10, Thermo Scientific).

### 4.3. Optic Nerve Crush

Mice were anesthetized by xylazine and ketamine based on their body weight (0.01 mg xylazine/g + 0.08 mg ketamine/g). Optic nerves on both sides were sequentially exposed intraorbitally and crushed with a jeweler’s forceps (Dumont #5; Fine Science Tools, Heidelberg, Baden-Württemberg, Germany) for 2 s, approximately 1 mm behind the eyeball. Care was taken to avoid damaging the underlying ophthalmic arteries. Erythromycin eye ointment was applied to protect the cornea after surgery.

### 4.4. Intravitreal Injection

Mice were anesthetized by xylazine and ketamine based on their body weight (0.01 mg xylazine/g + 0.08 mg ketamine/g). For intravitreal injection of chemical compounds or PBS control, a micropipette was inserted into the eyeballs of 6-weeks-old mice just behind the ora serrata and advanced into the vitreous chamber to avoid damage to the lens. After ONC injury, approximately 2 μL of the vitreous was removed before injection of 2 μL of chemical compounds or PBS control into the vitreous chamber. The chemical compounds for injection were as follows: 10 μM naringenin, 10 μM nobiletin, and 5 μM hesperidin. The drug injections were performed right after crush injury.

### 4.5. Cell Cultures and Treatments

HEK293T cells were cultured in Dulbecco’s Modified Eagle’s medium (DMEM) (Cat# 11320033, GIBCO) supplemented with 10% fetal bovine serum (FBS) (Cat# 10091148, GIBCO) at 37 °C in a 5% CO_2_ incubator. Twenty-four hours after plating, the cells were first treated with 500 mM of sorbitol for 30 min and then with sequential concentrations of each compound for 1 h. Finally, the cells were suspended, washed with ice-cold PBS, and centrifuged at 3000 rpm for 10 min.

### 4.6. Cell Toxicity Analysis of Compounds

HEK293T cells were cultured in DMEM supplemented with 10% FBS in 96-well plates at 37 °C in a 5% CO_2_ incubator. Twenty-four hours after plating, the cells were treated with a series of concentrations of each compound for 24 h and then treated with MTS-based CellTiter 96^®^ AQueous assay kits (Cat# G1111, Promega, Madison, WI, USA) and finally measured by the absorbance at 490 nm in 96-well plate reader which is directly proportional to the number of living cells in culture. Each compound concentration was assessed with 6 replicates.

### 4.7. Western Blot

The cell pellets were suspended and lysed in radio-immunoprecipitation assay buffer (50 mM Tris HCl with pH 8.0, 150 mM NaCl, 1% NP-40, 0.5% sodium deoxycholate, 0.1% SDS, 5 mM sodium pyrophosphate, 10 mM sodium fluoride, 1 mM sodium orthovanadate, and protease inhibitor cocktail) on ice for 30 min. The lysates were centrifuged at 12,000× *g* for 10 min, and supernatants were subjected to 10% SDS–polyacrylamide gel electrophoresis. After transfer onto a polyvinylidene difluoride (PVDF) membrane (Cat# IPVH00010, Merck Millipore, Billerica, MA, USA), the membranes were blocked with 5% milk, probed with primary and secondary antibodies, and exposed using an Amersham Imager 600 chemiluminescence imager (GE Healthcare, Boston, MA, USA). The band intensity was analyzed using ImageJ software (NIH, Bethesda, MD, USA). The primary antibodies used were rabbit anti-phospho-JUN and mouse anti-GAPDH.

### 4.8. Retina Immunohistochemistry Staining and RGC Counting

Retinas were dissected out from 4% paraformaldehyde-fixed eyes and washed extensively in PBS before blocking in staining buffer (10% normal goat serum and 2% Triton X-100 in PBS) for 30 min. Antibodies were diluted in the same staining buffer. Floating retinas were incubated with primary antibodies overnight at 4 °C and washed three times for 30 min each with PBS. Secondary antibodies were then applied and incubated for 1 h at room temperature. Retinas were again washed three times for 30 min each with PBS before a cover slip was attached with Fluoromount-G.

For RGC counting, three fields were randomly collected from peripheral regions of each retina to estimate RGC survival. The investigator who counted the cells was blinded to the preparing and imaging of the retina samples. The percentage of RGC survival was calculated as the ratio of surviving RGC numbers of injured eyes compared to that of uninjured naive eyes.

### 4.9. Statistical Analyses

Data are presented as mean ± SEM, and Student’s *t*-test was used for two-group comparisons, and one-way ANOVA with Bonferroni’s multiple comparisons test was used for multiple comparisons. *p* ≤ 0.05 was considered statistically significant. Animal experiments were repeated two times and HEK293T cell experiments were performed with 4–6 replicates.

## Data Availability

The datasets used and/or analyzed during the current study are available through the corresponding author upon reasonable request.
